# A conceptual framework for understanding stress-induced physiological and transgenerational effects on population responses to climate change

**DOI:** 10.1093/evlett/qrad037

**Published:** 2023-09-29

**Authors:** Ondi L Crino, Russell Bonduriansky, Lynn B Martin, Daniel W A Noble

**Affiliations:** College of Science and Engineering, Flinders University, Bedford Park, SA, Australia; Division of Ecology and Evolution, Research School of Biology, The Australian National University, Canberra, ACT, Australia; Evolution and Ecology Research Centre, School of Biological, Earth and Environmental Sciences, University of New South Wales, Sydney, NSW, Australia; Global Health and Infectious Disease Research Center and Center for Genomics, University of South Florida, Tampa, FL, United States; Division of Ecology and Evolution, Research School of Biology, The Australian National University, Canberra, ACT, Australia

**Keywords:** epigenetics, glucocorticoids, intergenerational effects, maternal effects, population resilience, temperature

## Abstract

Organisms are experiencing higher average temperatures and greater temperature variability because of anthropogenic climate change. Some populations respond to changes in temperature by shifting their ranges or adjusting their phenotypes via plasticity and/or evolution, while others go extinct. Predicting how populations will respond to temperature changes is challenging because extreme and unpredictable climate changes will exert novel selective pressures. For this reason, there is a need to understand the physiological mechanisms that regulate organismal responses to temperature changes. In vertebrates, glucocorticoid hormones mediate physiological and behavioral responses to environmental stressors and thus are likely to play an important role in how vertebrates respond to global temperature changes. Glucocorticoids have cascading effects that influence the phenotype and fitness of individuals, and some of these effects can be transmitted to offspring via trans- or intergenerational effects. Consequently, glucocorticoid-mediated responses could affect populations and could even be a powerful driver of rapid evolutionary change. Here, we present a conceptual framework that outlines how temperature changes due to global climate change could affect population persistence via glucocorticoid responses within and across generations (via epigenetic modifications). We briefly review glucocorticoid physiology, the interactions between environmental temperatures and glucocorticoid responses, and the phenotypic consequences of glucocorticoid responses within and across generations. We then discuss possible hypotheses for how glucocorticoid-mediated phenotypic effects might impact fitness and population persistence via evolutionary change. Finally, we pose pressing questions to guide future research. Understanding the physiological mechanisms that underpin the responses of vertebrates to elevated temperatures will help predict population-level responses to the changing climates we are experiencing.

## Introduction

In the last century, humans have dramatically changed the global climate, resulting in a rapid loss of biodiversity that has been described as the sixth mass extinction (Bellard et al., 2012; Ceballos & Ehrlich, 2018; Ceballos et al., 2015). Populations can respond to changing climates by shifting their ranges or phenologies or through other adaptations, while others go extinct (Pecl et al., 2017; Scheffers et al., 2016; Sinervo, 2010). The ability to predict how populations respond to changing climates is a prerequisite to developing effective conservation and management plans (Moritz & Agudo, 2013). Yet, our ability to understand and predict population responses to global climate change is hindered by limited knowledge in areas such as (a) the physiological mechanisms that promote or limit phenotypic responses to changing environmental conditions (Johnston et al., 2019), (b) whether and how environmentally induced physiological responses can be transmitted across generations (i.e., trans- and intergenerational effects, see Glossary), and (c) whether and how trans- and intergenerational responses affect adaptation and population resilience. Physiological responses link changes in the environment to whole organism phenotype and individual fitness and, ultimately, can affect demography, community structure, population resilience, and evolutionary dynamics (Kimball et al., 2012; Laughlin et al., 2020). Thus, understanding the physiological mechanisms that mediate organismal responses to global climate change, and the costs associated with such responses, is critical for understanding population- and species-level outcomes.

Higher temperatures and more frequent exposure to elevated temperatures are key outcomes of global climate change that will impact organismal physiology. Glucocorticoids are well-studied hormones that are part of the vertebrate physiological stress response and are expected to play an important role in determining vertebrate responses to elevated temperatures associated with global climate changes (Angelier & Wingfield, 2013; Mentesana & Hau, 2022; Meylan et al., 2012). Comparative work shows connections between temperature and glucocorticoid physiology (e.g., Alfonso et al., 2021; de Bruijn & Romero, 2018; Jessop et al., 2016; Racic et al., 2020). These patterns can help us predict how climate-driven changes in glucocorticoid physiology will affect fitness. A separate area of research has highlighted the importance of transgenerational phenotypic plasticity in shaping species responses to global climate change (Donelan et al., 2020; Donelson et al., 2018; Sgro et al., 2016). However, we currently lack a conceptual framework that unifies these levels of analysis to help us understand how temperature-driven effects on glucocorticoids could change within and across generations to impact life history, population, and evolutionary dynamics.

Here, we take steps toward building such a conceptual framework. To do so requires that we make simplifying assumptions about glucocorticoid physiology. We believe that even a simplified framework will provide a starting point to more rigorously explore previous ideas using targeted theoretical, comparative, and empirical studies. Additionally, for the sake of simplicity, we focus on the effects of elevated temperatures associated with global climate change on glucocorticoid physiology but recognize that global climate change can result in many altered climate patterns. We believe that our framework has broad applicability to a range of climate variables associated with global climate change given the role of glucocorticoids in regulating animal responses to environmental conditions. We begin by discussing how glucocorticoid physiology regulates vertebrate responses to environmental disturbances, such as elevated temperatures, and how such responses can affect individual fitness. Next, we discuss how glucocorticoid responses can be transmitted across generations to affect population persistence and adaptive evolution. Finally, we discuss how future studies addressing the role of glucocorticoids in regulating species responses to global climate changes can fill current knowledge gaps.

## Glucocorticoids regulate vertebrate responses to environmental perturbations such as extreme temperatures and affect individual fitness

Glucocorticoids are steroid hormones that regulate metabolic function at baseline levels and play an important role in the vertebrate “stress” response (Sapolsky et al., 2000). Following disturbances or threats, vertebrates increase glucocorticoid secretion via activation of the hypothalamic–pituitary–adrenal/interrenal axis (HPA/HPI axis, depending on taxonomic group). Elevated glucocorticoid levels promote physiological and behavioral responses (e.g., metabolic changes, vigilance behavior) that allow animals to cope with perturbations such as food limitation and predation pressure (Romero, 2004; Sapolsky et al., 2000; Wingfield & Kitaysky, 2002). Short-term or acute exposure to glucocorticoids is thought to promote adaptive responses to disturbances (McEwen & Wingfield, 2003; Wingfield et al., 1998). In contrast, sustained or chronic exposure to elevated glucocorticoids has been associated with phenotypic effects that are assumed to have negative effects on fitness, such as suppression of the immune system, reduced body condition, and neuronal cell death (Romero et al., 2009).

Elevated glucocorticoid levels have been linked to weather events and environmental temperatures (de Bruijn & Romero, 2018; Jessop et al., 2016; Vitousek et al., 2022; Wingfield, 2013) and are hypothesized to play an important role in promoting adaptive organismal responses to changes in temperature associated with global climate change (Hau et al., 2022; Mentesana & Hau, 2022; Ruuskanen et al., 2021). Glucocorticoids can induce physiological and behavioral changes that enable animals to acclimate to changes in temperatures (and thus increase survival). For example, elevated levels of glucocorticoids could help animals cope with high temperatures by increasing the availability of energy needed to thermoregulate via panting, evaporative water loss, facultative hyperthermia, and other heat-dissipating strategies (McKechnie & Wolf, 2019; Tattersall et al., 2012). In support, a study of 94 bird species found a positive association between thermoregulatory costs (estimated by biophysical models) and baseline glucocorticoid levels (Rubalcaba & Jimeno, 2022). Glucocorticoids may also promote behaviors and affect activity budgets in ways that allow animals to cope with elevated temperatures (Mentesana & Hau, 2022).

Acute elevation of glucocorticoid levels is thought to induce behaviors and physiological responses that promote self-maintenance and survival at the expense of reproduction (Wingfield et al., 1998). Mechanistically, elevated levels of glucocorticoids can suppress the production of hormones needed for reproduction and, consequently, reduce reproductive and parental behaviors (Angelier & Chastel, 2009; Lendvai & Chastel, 2010; Lynn et al., 2010; Wingfield & Sapolsky, 2003). In this way, persistently elevated levels of glucocorticoids in response to temperature changes can decrease individual fitness by decreasing lifetime reproductive success. Additionally, sustained exposure to glucocorticoids has been associated with increased production of reactive oxygen species and oxidative damage (e.g., telomere attrition; reviewed in Angelier et al., 2018; Costantini et al., 2011; Hau et al., 2015), suggesting that long-term exposure to glucocorticoids may affect net fitness by decreasing life span.

**Figure 1.**
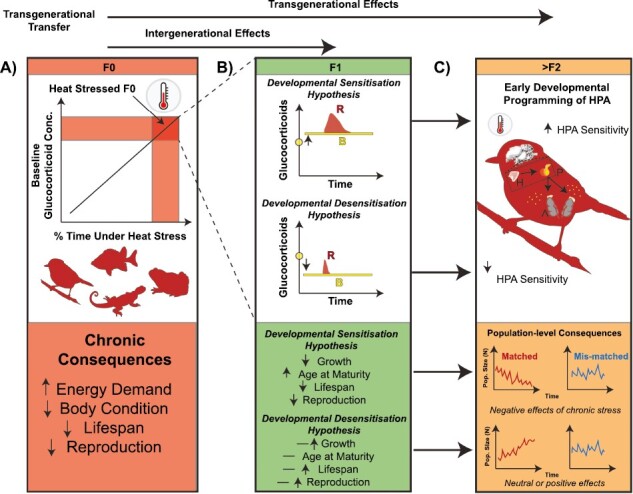
Framework for understanding the effects of thermal stress on glucocorticoid physiology, life history, and fitness across generations. (A) Repeated acute or chronic exposure to heat stress is expected to result in elevated glucocorticoid levels because chronic thermal stressors increase baseline glucocorticoid levels (e.g., Jessop et al., 2016; Mentesana & Hau 2022; Narayan & Hero 2014). Such effects are predicted to increase energy demand, which will reduce body condition (assuming resources are limited), and compromise reproduction and survival probability over the long term; (B) focusing on offspring from F_0_ adults exposed to thermal stress, as indicated by the dashed lines to F_1_ box, intergenerational effects (e.g., maternal effects) are expected to result in either (i) sensitization of the HPA (i.e., Developmental Sensitization Hypothesis) or (ii) desensitization of HPA responses (i.e., Developmental Desensitization Hypothesis) in F_1_ offspring. The yellow dot on the y-axis in each figure represents the hypothetical GC concentration of F_0_ adults. Under the Developmental Sensitization Hypothesis, baseline glucocorticoid levels in the F_1_ are expected to increase because the offspring HPA is more reactive to stressors (i.e., higher peak responses and slower return to baseline—denoted by differences in red response curve “R”). If offspring are exposed to repeated thermal stressors (i.e., an environment like their parents), repeated acute responses lead to elevated glucocorticoid levels (denoted by higher baseline “B” glucocorticoid levels) and we expect reduced growth, slower age at maturity, and decreased life span and reproduction in offspring. Under the Developmental Desensitization Hypothesis, maternal effects are expected to cause the HPA axis in offspring to become less responsive to stressors (i.e., reduced peak glucocorticoid levels and faster return to baseline). (C) Transgenerational effects of glucocorticoids can be long lasting (i.e., epigenetic, or behavioral perpetuation—across many generations) or transient (hormonal, resource driven—across one or a couple generations) with their effects on population persistence depending on environmental predictability (i.e., matched or unmatched environments). Under the Developmental Sensitization Hypothesis, positive feedback could occur, such that responses are compounded across generations under repeated environmental stress. The HPA axis (H = Hypothalamus; P = Pituitary gland; A = Adrenal glands) is expected to become more sensitive for a larger fraction of the population. In this scenario, reduced population growth is expected because of the negative effects of chronically elevated glucocorticoids on life-history traits, affecting survival and reproduction,increases the probability of population extinction. In contrast, developmental desensitization could reduce the sensitivity of the HPA axis in subsequent generations, reducing glucocorticoid levels and exerting a neutral or beneficial effect on organism fitness.

Temperature extremes and fluctuations have been linked to elevated glucocorticoid levels across taxonomic groups in wild animals (Bonnet et al., 2013; Huber et al., 2003; Racic et al., 2020; Shahjahan et al., 2017), suggesting that chronic exposure to elevated temperatures will lead to greater cumulative exposure to glucocorticoids (Wingfield & Sapolsky, 2003; Monaghan, 2014; Jessop et al., 2016; Mentesana & Hau, 2022; Figure 1). The phenotypic responses and associated fitness consequences of glucocorticoids are affected by physiological variables such as the magnitude of glucocorticoid secretion, hormone receptor density (i.e., mineralocorticoid and glucocorticoid receptors), the availability of transport proteins (i.e., corticosteroid binding globulin), and metabolic enzymes (e.g., 11β-HSD), among other traits (Sapolsky et al., 2000; Lema & Kitano, 2013). Flexibility in these different components of glucocorticoid physiology may enable animals to match phenotypic responses to prevailing environmental conditions (and thus increase individual fitness), ultimately allowing populations to adapt to local conditions (Zimmer et al., 2022). However, the fitness effects of changes in glucocorticoid physiology are likely to be highly context and species specific with both adaptive and maladaptive effects possible (Bonier et al., 2009a; Breuner et al., 2008; Schoenle et al., 2018).

## Glucocorticoids can have intergenerational effects on offspring fitness

Beyond their effects on individual fitness, glucocorticoids have also been implicated as key mediators of intergenerational (F_1_) effects (McCormick et al., 2017; Zimmer et al., 2017). Developing animals can be directly exposed to maternal glucocorticoids through embryonic exposure in ovo or in utero or postnatally via breast milk (e.g., Groothuis et al., 2019; Ruuskanen & Hsu, 2018; Sopinka et al., 2017). Additionally, developing animals can be indirectly exposed to maternal glucocorticoids when they affect maternal phenotype in a way that causes offspring to increase their own endogenous production of glucocorticoids (e.g., via changes in maternal care). Exposure to elevated glucocorticoid levels during development can influence the lifelong function of the HPA axis in the F_1_ generation, resulting in higher baseline glucocorticoid levels and increased glucocorticoid secretion in response to disturbances and stressors (referred to here as the “Developmental Sensitization Hypothesis”; Figure 1; Catalani et al., 2011; Crino et al., 2022; Spencer et al., 2009; Thayer et al., 2018). A recent meta-analysis found an overall positive relationship between prenatal glucocorticoid exposure and offspring glucocorticoid responses across 14 vertebrate species, suggesting that increased prenatal sensitivity to glucocorticoids is probably widespread (Thayer et al., 2018). Alternatively, embryonic exposure to glucocorticoids may result in desensitization of the HPA axis, making offspring less responsive to environmental stressors (referred to here as the “Developmental Desensitization Hypothesis”; Figure 1; Ho & Burggren, 2010; Meylan et al., 2012). Both hypotheses are plausible but are likely to have different fitness consequences for offspring (detailed in Figure 1B).

## Glucocorticoids as mediators of transgenerational phenotypic plasticity

Glucocorticoids have been implicated as key mediators of transgenerational effects, such that conditions that elevate glucocorticoids in the parental generation can affect the development and phenotypes of future generations (Kraft et al., 2021; McCormick et al., 2017; Zimmer et al., 2017). Depending on the taxa and parent exposed, transgenerational effects can be detected in F_2_ (grand-offspring) or F_3_ descendants and beyond (e.g., Kraft et al., 2021; Moisiadis et al., 2017). Glucocorticoids can drive such transgenerational effects via changes in the expression of genes related to histone methylation and other epigenetic modifications (Box 1; Moisiadis & Matthews, 2014; Mousseau & Fox, 1998). For example, exposure to stressors during development can result in epigenetic modifications of glucocorticoid receptor genes (Ahmed et al., 2014; Francis et al., 1999; Liu et al., 1997; Weaver et al., 2004). Glucocorticoid levels are regulated through negative feedback of glucocorticoids and other hormones associated with the HPA axis (Sapolsky et al., 2000). Reduced expression of mRNA and proteins associated with glucocorticoid receptors in the hippocampus slows the attenuation of the stress response, resulting in longer glucocorticoid responses to disturbances (De Kloet et al., 1998; Lucassen et al., 2013). For example, rat pups that receive less maternal care require more time for stress-induced glucocorticoid levels to return to baseline levels resulting in prolonged HPA responses to stressors (Francis & Meaney, 1999; van Oers et al., 1998). Changes in HPA responses of F_1_ offspring are mediated by methylation within the promoter region of the hippocampal glucocorticoid receptor (GR1_7_) gene of offspring, with greater methylation resulting in lower receptor expression (Weaver et al., 2004). Methylation patterns can be transmitted behaviorally from mothers to their offspring, such that offspring that receive less maternal care supply their own offspring with less care, resulting in the perpetuation of stress responses across multiple generations (i.e., >F_2_) (Champagne, 2008; Francis et al., 1999; Weaver et al., 2004).

Box 1. **Epigenetics and transgenerational effects**

Environmental conditions can induce epigenetic modifications of the egg or sperm genome that influence the development of offspring and, in some cases, grand-offspring (F_2_) and beyond (Angers et al., 2020; Bell & Hellmann, 2019). Several epigenetic mechanisms have been implicated in transgenerational effects, including DNA methylation, histone modification, and small, noncoding RNAs (Skinner, 2014a; Skinner et al., 2010). DNA methylation and histone modification involve molecular changes to DNA segments that affect the level and pattern of gene transcription (Zhang & Pradhan, 2014). Noncoding RNAs can modulate gene expression by gene silencing and by affecting the mechanisms of DNA methylation and histone modification (Costa, 2008). Transmission of environment-induced epigenetic variants through the germline (“transgenerational epigenetic inheritance”) has been reported in nematodes, flies, rodents, and other animals, although the molecular mechanisms involved appear to vary among taxonomic groups (Fitz-James & Cavalli, 2022).

Inheritance of epigenetic modifications can be advantageous in both stable and fluctuating environments if inheritance enhances the match between offspring phenotype and the environment that offspring are likely to experience (Rivoire & Leibler, 2014). Epigenetic factors can also mediate the transfer of parental body condition to offspring, enabling parents in good condition to enhance the viability of their offspring in any environment (Bonduriansky, 2021; Bonduriansky & Crean, 2018; Qvarnstrom & Price, 2001). However, epigenetic modifications could also be pathological and reduce offspring performance (Bonduriansky, 2021).

Theory suggests that epigenetic inheritance, and other forms of nongenetic inheritance such as parental effects, could play an important role in adaptive evolution (Bonduriansky & Day, 2018; Klironomos et al., 2013). In populations where additive genetic variance is low, variation in heritable epigenetic and other nongenetic factors could contribute substantially to the net pool of heritable variation, and experimental evidence suggests that such factors could respond to natural selection and drive rapid changes in important traits (Cropley et al., 2012). Phenotypic change driven by selection on nongenetic factors could enable populations to keep pace with rapid environmental change (Klironomos et al., 2013). Genetic change is expected to occur more slowly, and potentially result in the replacement of nongenetic adaptations with more stable genetic factors once the population mean phenotype has approached the new fitness peak (Pal & Miklos, 1999). However, in a continually changing environment (e.g., one with increasing or fluctuating temperatures), nongenetic factors could continue to drive population responses.

## Glucocorticoid-mediated effects on population persistence

Glucocorticoids are predicted to affect population resilience by impacting individual life history and fitness (Figure 1; Meylan et al., 2012). However, the effects of glucocorticoids on individuals, and consequently population resilience, are likely to depend on many factors including sex, age, and the duration of exposure to elevated glucocorticoid levels (i.e., chronic vs. acute exposure; Angelier & Wingfield, 2013; Mentesana & Hau, 2022). For example, elevated glucocorticoid levels could affect the age structure of populations if they result in high offspring mortality and slow maturation. Given that each sex may be affected differently by glucocorticoids (e.g., Iqbal et al., 2012), acute and chronic effects also have the potential to alter population sex ratios (Crespi et al., 2013). Population-level consequences of glucocorticoids will also depend on environmental predictability, how thermal environments shape HPA axis responses (Figure 1), and the transiency of such responses within and across generations. For example, high resource availability could dampen the costs of elevated HPA axis responses because well-fed animals can maintain body condition despite frequent thermal stressors. If the environment of parents accurately predicts that of the offspring and the HPA axis becomes desensitized, then adaptative transgenerational plasticity can enhance offspring fitness and promote population persistence ). For example, red squirrel (*Tamiasciurus hudsonicus*) mothers experiencing high conspecific density have heightened glucocorticoid levels, leading to intergenerational effects that result in a faster pup growth rate and an increased probability of recruitment (Dantzer et al., 2013). Adaptive transgenerational plasticity in glucocorticoid responses could allow populations to persist or even increase in the face of environmental stress (Figure 1C) if dampened HPA responses alleviate the long-term costs of chronically elevated glucocorticoid levels without decreasing immediate survival. Adaptive transgenerational changes in glucocorticoid physiology could also result in phenotypes adapted for a generalized set of environmental stressors, which may be adaptive in multistressor environments that are likely to coincide with extreme climate change (Liebl & Martin, 2012; Martin & Liebl, 2014; Potticary & Duckworth, 2020). In contrast, reduced population growth is expected if the HPA axis is sensitized across generations, particularly if parental and offspring environments are matched (Figure 1C). Repeated stressors experienced by future generations that have oversensitive HPA axes could compromise reproduction and survival because of the negative fitness effects of chronically elevated glucocorticoids (see above).

## Glucocorticoid physiology, transgenerational plasticity, and evolutionary adaptation to climate change

**Figure 2.**
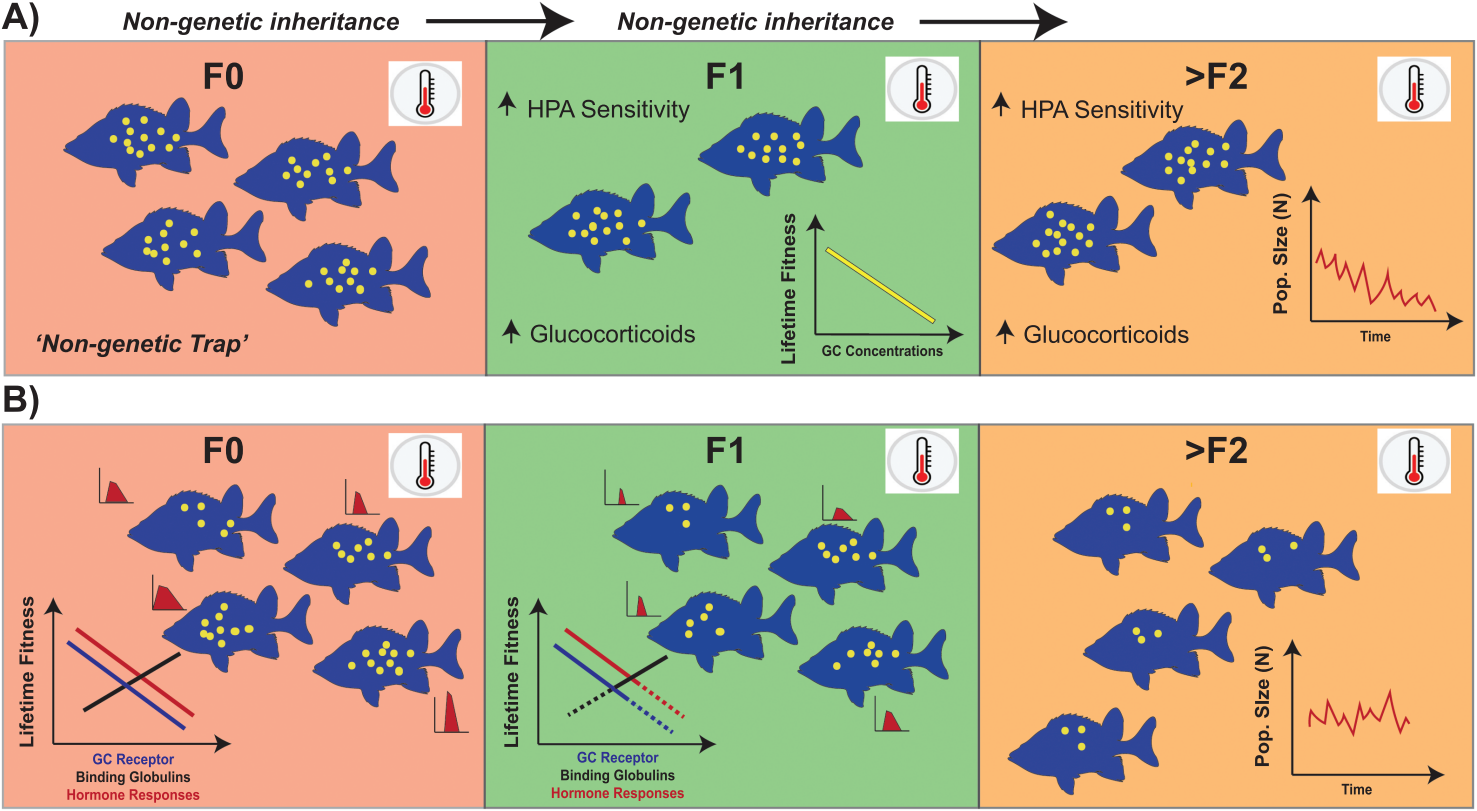
Hypothesized pathways in which nongenetic and genetic variation might affect the capacity of populations to evolve in response to the costs of chronically elevated glucocorticoids. Each colored box corresponds to the generation (“light red” = “F_0_”; “light green” = “F1”; and “light orange” = “>F2”). “Yellow” dots signify GC concentrations within each fish. (A) Hypothetical situation where sensitized HPA responses (in the absence of genetic variation) are likely to be propagated across generations through nongenetic inheritance (e.g., epigenetic, behavioral propagation, etc.), such that the sensitivity of HPA axis is increased, leading to lifetime fitness costs (inset graph in F1 generation). Such effects are predicted to lead to population decline as populations are caught in a “Non-genetic Trap” (O’Dea et al., 2016). (B) Genetic and nongenetic (epigenetic) variation impacting variation in key factors involved in HPA responses (i.e., glucocorticoid/mineralocorticoid receptor density and affinities, variation in binding globulins, and GC response curves) result in some individuals being less sensitive to thermal stressors (Meylan et al., 2012; Taborsky et al., 2021). Small inset burgundy-filled curves are GC response curves for each fish. The height of each curve for each fish represents the maximal GC response and the width the clearance time of GC’s. Selection in F_0_, results in the F_1_ population evolving to exhibit different glucocorticoid responses such that variants controlling the expression of GC traits with reduced fitness are selected against (“dashed” parts of lines in F_1_ inset). Natural selection may also act on the HPA axis to promote adaptive plasticity or endocrine flexibility (sensu Zimmer et al. 2018), which could be driven by genetic or nongenetic mechanisms (i.e., epigenetic modifications). Changes in GC plastic responses in the F1 generation is predicted to result in lower peak responses and/or faster clearance times.

Predicting the consequences of transgenerational glucocorticoid-mediated effects on evolutionary dynamics will be challenging. Currently, there are no clear examples that provide insight into how selection on genetic and nongenetic factors might be interwoven to shape the evolution of glucocorticoid responses. However, based on research across disciplines, we can generate predictions in relation to how thermal stress will impact the evolution of HPA axis function (Figure 2). First, if we assume that HPA axis function is influenced by developmental conditions and that there are negative fitness costs to chronically elevated glucocorticoids, then we would predict that existing physiological mechanisms that regulate glucocorticoid secretion will evolve to function in a more adaptive way (e.g., such that high glucocorticoid levels are avoided or limited in duration; Figure 2B). Alternatively, if temperature fluctuations increase in amplitude but continue to exhibit autocorrelation across time, maternal effects could similarly evolve to maintain adaptive functionality by minimizing the transmission of glucocorticoids in eggs, milk, or the intrauterine environment (Dey et al., 2016; Furrow & Feldman, 2014). Third, acute or chronic increases in glucocorticoid levels could induce novel patterns of DNA methylation, chromatin structure, or noncoding RNA synthesis in the soma and germline (Gudsnuk & Champagne, 2012; Kundakovic & Jaric, 2017; Maccari et al., 2003; Palma-Gudiel et al., 2015). If such stress-induced epigenetic factors can be transmitted across generations (e.g., Crews et al., 2012; Skinner, 2014b), these epigenetic variants could respond to selection (Cropley et al., 2012), potentially resulting in adaptive “epigenetic evolution” (Figure 2C). Theory suggests that epigenetic evolution can occur rapidly in novel environments because similar changes can be induced in multiple individuals at the same time and might be particularly important when additive genetic variance is very low (Kilvitis et al., 2017; Rollins et al., 2015). However, epigenetic evolution can only lead to stable, cumulative change across generations if induced epigenetic changes are relatively stable over multiple generations (Furrow, 2014).

## Future research directions

Currently, few studies explicitly link the developmental and transgenerational effects of glucocorticoids to population and evolutionary dynamics despite a recognition of their importance at the individual level (Boonstra et al., 1998; Crespi et al., 2013; Dantzer et al., 2013). Additionally, few studies in vertebrates have measured how elevated glucocorticoid levels brought about by high temperature influence investment in reproduction versus survival to affect lifetime fitness. Here, we outline some promising new research directions that could help clarify how developmental and transgenerational effects on glucocorticoids impact ecological and evolutionary dynamics.

### Testing links between temperature, glucocorticoids, and fitness

In recent years, a growing body of research has reported links between increasing global temperatures and fitness across vertebrate species (e.g., Dayananda et al., 2016; Paniw et al., 2022; van de Ven et al., 2020). Other studies have linked elevated temperatures to changes in glucocorticoid physiology (e.g., Chadwick & McCormick, 2017; Moagi et al., 2021; Xie et al., 2017). However, empirical studies in vertebrates are needed that explicitly test links between temperature, glucocorticoid physiology, and lifetime fitness. Admittedly, such studies are logistically challenging and perhaps only feasible in study systems with short generation times that can be maintained in captivity. However, experiments that manipulate environmental temperatures and track the long-term effects on physiology and fitness components will uncover the potential role of glucocorticoids in shaping individual fitness in response to global climate change.

### Integrating modeling approaches that link individual variation in fitness to population persistence

New modeling approaches, such as integral projection models (Coulson, 2012), that allow for individual-level traits, such as circulating glucocorticoids in parents (or grandparents) and offspring to be integrated into fitness functions (survival and fecundity) can be used to predict population dynamics. Models can also be developed to highlight sensitive life-history stages and sex-dependent effects. These models can explicitly decompose genetic and nongenetic contributors to phenotypes, enhancing our understanding of how these factors interact to affect both population (Creel et al., 2013) and evolutionary dynamics (Coulson et al., 2021; Simmonds et al., 2020). Developing such models will likely only be possible for short-lived species or those with long-term data. Nonetheless, they show great promise in linking glucocorticoid physiology, potential transgenerational effects, and ecological and evolutionary outcomes.

### Understanding the adaptive and maladaptive nature of chronic elevation in glucocorticoid levels

While chronic elevation of glucocorticoid levels is likely to impose fitness costs, fitness effects are likely to be highly context and species specific (Bonier et al., 2009a; Breuner et al., 2008; Schoenle et al., 2018). For example, negative relationships between baseline glucocorticoid levels and fitness components have been identified during some life-history stages such as reproduction (e.g., Bonier et al., 2009b), while positive relationships have been described during other life-history stages such as development and postreproduction (e.g., Bonier et al., 2009b; Rivers et al., 2012). Here, we have assumed that developmentally sensitized glucocorticoid responses are typically maladaptive, but this need not always be the case. For example, adaptive effects could result by promoting responses that allow animals to cope with acute stressors more quickly. In fact, it is not always the case that higher levels of glucocorticoids result in negative long-term fitness effects (Boonstra, 2013; Dantzer et al., 2013). Understanding the trade-offs between survival versus long-term reproductive success will be critical to understanding how ecological and evolutionary scenarios are likely to play out.

### Importance of environmental stability and persistence of nongenetic effects

Theoretical modeling of stress–response curves suggests that the environmental predictability of stressors is pivotal to how they evolve (Taborsky et al., 2021). The stability of epigenetic variants (such as those induced by glucocorticoid exposure) across generations remains poorly understood (Bertozzi et al., 2021; Johannes et al., 2009). Consequently, how stress responses should change with the inclusion of inter- and transgenerational effects is not yet clear, and theoretical models have not been rigorously tested empirically. Likewise, it remains unclear in most cases to what extent induced epigenetic changes are independent of the genotype (Richards 2006; Villicana & Bell, 2021). Evolutionary models, combined with comparative and empirical tests of their predictions and assumptions, hold great promise in illuminating the role of inter- and transgenerational effects on stress response evolution, and we encourage such approaches in the future (as discussed by Taborsky et al., 2021).

## Conclusions

Glucocorticoids are expected to play an important role in mediating the responses of vertebrates to global climate change. However, given the highly pleiotropic effects of glucocorticoids, temperature-dependent changes in glucocorticoid levels and stress–response curves are likely to have broad effects on behavior and life history with consequences on individual fitness and population persistence. Elevated glucocorticoids can induce phenotypic responses that impact not only the exposed individuals but also their descendants, via maternal transfer (i.e., intergenerational effects) or transgenerational effects transmitted to grand-offspring. The net effect of altered glucocorticoid levels on population persistence and adaptation will therefore depend on the balance of adaptive and maladaptive effects as well as the persistence of any transgenerational effects. In combination with quantitative modeling approaches, empirical studies that manipulate ambient temperature in parents and assess effects on offspring and grand-offspring in both unmatched (to quantify persistence) and matched environments (to quantify cumulative effects) would be illuminating. Future studies that span vertebrate taxonomic groups will be particularly relevant as some life-history traits will increase susceptibility to global climate change (e.g., generation time, mode of thermoregulation, etc.).

## Date and code availability

No new data were generated or analyzed in support of this research.

## Glossary

**DNA methylation:** The addition of a methyl group (-CH^3^) to the cytosine ring of DNA. DNA methylation can change the likelihood that a sequence of DNA is transcribed without changing the DNA sequence. DNA methylation typically acts to reduce gene expression when located in gene promoter regions.

**Epigenetic modifications:** Changes in gene expression that are not caused by changes to the primary DNA sequence. Epigenetic modifications can involve mechanisms such as DNA methylation, histone modification, and small noncoding RNAs.

**Glucocorticoids:** Steroid hormones of vertebrates that regulate metabolic processes and play a role in the stress response. Baseline levels of glucocorticoids refer to endogenous levels of hormones when animals are undisturbed or not acutely challenged by an environmental factor. Peak levels of glucocorticoids follow an acute disturbance and promote physiological and behavioral changes that allow animals to cope with challenges or stressors. The phenotypic responses to glucocorticoids are often discussed in terms of *acute* versus *chronic* exposure. Acute exposure to glucocorticoids is generally thought to span minutes to hours and to induce adaptive physiological and behavioral responses. In contrast, chronic exposure to glucocorticoids spans days to weeks and results in seemingly deleterious phenotypic effects that are thought to reduce fitness (but see Boonstra, 2013). Corticosterone is the main glucocorticoid that is produced by birds, reptiles, amphibians, and rodents. Cortisol is the main glucocorticoid produced by fish and mammals (except rodents).

**Histone modifications:** Changes in the physical properties of chromatin (a cellular constituent that packages DNA into chromosomes) that affect accessibility of DNA to transcription factors. Examples include histone acetylation, phosphorylation, and methylation among others.

**Hypothalamic–pituitary–adrenal/interrenal (HPA/HPI) axis:** A neuroendocrine pathway that regulates the release of glucocorticoid hormones from the adrenal or interrenal glands (depending on taxonomic group; HPI in teleost fish and HPA in all other vertebrates).

**Intergenerational effects:** The transmission or influence of traits from parents (F_0_) to their offspring (F_1_) via nongenetic factors.

**Maternal effects:** Phenotypic traits or characteristics elicited in one generation (F_1_) that depend on the mother’s (F_0_) phenotype. Maternal effects are not included within the scope of transgenerational effects because developing F_1_ offspring directly experience the maternal environment that elicits the response. Maternal effects are an example of intergenerational effects.

**Phenotypic plasticity:** The ability of a single genotype to produce alternative phenotypes in response to environmental conditions. Phenotypic plasticity can be adaptive, maladaptive, or neutral in regard to an individual’s fitness. Adaptive phenotypic plasticity can occur when past or current selection promotes phenotypic changes that increase tolerance and, hence, fitness across multiple environments or in response to environmental changes. In contrast, maladaptive phenotypic plasticity occurs when phenotypic responses move individuals away from optimal responses due to factors such as physiological or genetic constraints.

**Population persistence:** The probability that a population will persist to a given point in time. Population persistence is often used to describe the long-term viability of a population and can depend on factors such as genetic diversity, the minimum viable population size, and habitat availability and quality.

**Small noncoding RNAs:** Short RNA strands that are not translated into protein but that can affect gene expression by transcription interference or other processes.

**Stress:** Stress commonly refers to the biological responses that occur when a vertebrate perceives a threat or challenge to its well-being. Stress responses are often discussed in relation to increased production of glucocorticoids. The use of the term “stress” and referring to glucocorticoids as “stress hormones” is contentious in the field of comparative endocrinology. Here, we use these terms for the sake of simplicity but acknowledge that vertebrate physiologists may prefer more nuanced definitions.

**Transgenerational effects:** The transmission or influence of traits across multiple generations such that changes in one generation (F_0_) can impact not only immediate offspring but also subsequent generations (F_2_ and beyond) via nongenetic factors.

**Transgenerational epigenetic inheritance:** The transmission of epigenetic modifications across generations through the germline. Note that epigenetic effects on offspring that are mediated by parental behavior or physiology rather than by epigenetic modifications of DNA in eggs or sperm are not usually included within the scope of transgenerational epigenetic inheritance but can be an example of inter- or transgenerational effects.
